# Selections

**Published:** 1892-01

**Authors:** 


### Selections.

The Modern Treatment of Syphilis.—Acting upon the general knowledge that if carefully used mercury scarcely ever does harm, and that it often in chronic maladies, whether syphilitic or not, acts beneficially, I have, in common I suppose with many others, for long been in the habit of prescribing mercury in cases of ataxia. Very frequently patients appear to be greatly benefited by it ; more especially the severity of the pains and the tendency to gastric crises appear to be mitigated. I must confess, however, that I have never had in any single case anything which might be vaunted as a cure. If I were to quote the cases in which white atrophy of the optic nerves has occurred as a complication, I am afraid I should be obliged to confess that they have all advanced to blindness in spite of the remedy. It has not, however, been so in those cases in which *ophthalmoplegia externa*, or paralysis of single muscles of the eyeball have been the complicating condition. In nearly all these great benefit has appeared to result from the long continued use of specifics. In these latter, the iodide of potassium, as well as mercury, is often very beneficial, whereas in locomotor ataxy itself I think I have often seen it prove definitely prejudicial, depressing the patient's vigor and making him feel low-spirited and miserable, without in any way mitigating his symptoms. In general paralysis of the insane, if there is a history of syphilitic antecedents, I would never omit the long-continued use of mercury. I have seen great benefit from its employment, and when we remember that its most common pathological condition is adhesion of the pia mater to the gray matter of the convolutions (implying the existence of a low form of inflammation), we may easily believe that if not required as a specific mercury may still very possibly be of use. It should be given as a long course of small doses. I have not as yet adverted to the treatment of syphilis in its inherited forms.

In infants, inunction is easily practiced in a variety of ways, and is usually very effectual. I have also found a solution of the bichloride, in small doses, a very efficient remedy, and not so liable to purge as the gray powder. If there is any evidence of bone disease, the iodide of potassium should be combined with it. If the symptoms are severe, and especially if the viscera are involved, infantile syphilis is undoubtedly a dangerous disease, and apt to terminate fatally by marasmus or convulsions. If, however, the specific is well borne, and the child passes favorably through the secondary stage, then I think there is, as a rule, very little danger of relapse; and a condition of good health may be expected until at a later period, say eight to fifteen years of age, the liability to keratitis, deafness, phagedænic affections of the throat, etc., may come on. These late manifestations of inherited taint occupy in reference to treatment a most exceptional position. Although we always prescribe specifics, they seldom or never appear to exercise any definite power. Keratitis will often run its course apparently almost uninfluenced, or the second eye may be attacked while the patient is under the remedies employed for the cure of the first. As regards the deafness, unless the remedies are used in its very earliest stage, I fear they very seldom prove of any value. It is certainly to be strongly urged in reference to both the deafness and the keratitis that mercury and iodides should be prescribed promptly and liberally, but we must be prepared to encounter much disappointment and to forego all hope of the rapid cures which the same remedies often effect in other conditions. It may be well that we should remember, in reference to this class of maladies, that they occur in those in whom probably the syphilitic virus has long ceased to be active, and who would be quite incapable of conveying the disease by contagion. They are tissue maladies, not the result of existing blood-poisoning. Hence, probably, in part, the impotence of mercury to manifest its specific power. There is no microbe left for it to kill.—*Jonathan Hutchinson, in The Practitioner.*

THE INDICATIONS FOR EARLY LAPAROTOMY IN APPENDICITIS.—Dr. W. W. Keen, of Philadelphia, agrees with those who hold that so-called "perityphlitic abscess" is almost always due to appendicitis (Reprint from the *Transactions of the Medical Society of the State of New York*, 1891.) For clinical purposes five forms of appendicitis are recognized by this surgeon: (1) a mild and non-perforative form, ending usually in resolution without suppuration; (2) perforative appendicitis followed by general peritonitis; (3) the most common form, in which the appendix is perforated and a local abscess forms more or less rapidly, which, if left to itself, ruptures externally or into a hollow viscus, and finally ends within a few weeks either in resolution or the death of the patient; (4) a class in which the abscess forms slowly and follows a chronic course, lasting for months before it either discharges or indicates an operation; (5) recurrent appendicitis, in which attacks are repeated at longer or shorter intervals. The first class of cases—that of mild appendicitis—may, Dr. Keen holds, be dismissed from consideration as not requiring operative treatment save in exceptional cases. Cases of the second class—in which perforative appendicitis is followed by acute general peritonitis—demand instant laparotomy. No cases in surgery—except, perhaps, those of hæmorrhage from large wounded vessels—require more prompt interference. The indications for immediate recourse to laparotomy in such cases are: brief symptoms of recent appendicitis, or of one or more recurrent attacks, followed by sudden excruciating pain over the whole abdomen, but most severe in the right iliac fossa, with the easily-recognized symptoms of general peritonitis and impending collapse. In cases in which perforative appendicitis takes for a time a mild course, and does not, until after an interval of some weeks, break out into general peritonitis, there is likely, in consequence of the deceptive mildness of the attack, to be much doubt as to the proper treatment. Dr. Keen thinks an exploratory operation should be undertaken when there is persistent pain and tenderness, with even slightly increased resistance without any tumor, and with possibly slight œdema and moderate ever. Many of these cases, Dr. Keen believes, may be included in the third class, in which an abscess—not, perhaps, of large size—has slowly developed, and at last has suddenly burst into the peritoneal cavity. Although there is occasionally much difficulty in distinguishing cases of this kind from those which run a continuously mild course and terminate in resolution, it is not impossible, it is held, to determine whether an abscess has formed or not. Dr. Keen relies mainly on local signs, which he regards as far superior to general constitutional symptoms. Even the temperature of the body may be a very deceptive guide, as this may be low while the local process is advancing towards a dangerous or fatal issue. He would lay it down as a rule, therefore, that even in mild cases, if the indications point even slightly towards suppuration, an early operation should be practiced. An exploratory operation, it is asserted, "carries with it less danger than the disease," and no patient should be allowed to run the risk of probable or possible rupture and of general peritonitis. The most reliable symptoms of a localized abscess are pain and tenderness in the right iliac fossa, especially marked at what is called "McBurney's point," which is situated one and a half or two inches from the anterior superior spine of the ilium, in a straight line towards the umbilicus, and œdema of the groin. If these signs be present, together with nausea and vomiting, rigidity of the abdominal wall on the right side, and fever, an exploratory operation should be performed on the second or third day. If no pus be found, the vermiform appendix should be sought for, and if, as Dr. Keen believes will almost uniformly be the case, it be found thickened, distended, occupied by a concretion, or otherwise abnormal, even without perforation, it should be tied and cut away.—*Brit. Med. Jour.*

---

VERY HIGH TEMPERATURE.—In the *Memphis Medical Monthly* for October, Dr. Heber Jones records his remarkable case of hyperpyrexia which excited no little interest and comment some months ago. Beginning at 109°, when first observed by Dr. Jones, the fickle mercury oscillated up and down the shaft of the thermometer all the way from 96½° to 157°. At this point the instrument was broken by the expansion of the mercury, which, unfortunately, leaves us in ignorance as to how high the temperature might have gone. It might possibly have eclipsed the remarkable case of Dr. Gailbraith, who once observed a temperature of 171°.

Dr. Jones' patient was a girl of fifteen, not of nervous temperament, who had always enjoyed average health. This illness began with a tonsillitis which was not severe, lasting about one week. The above extraordinary play of temperature then developed, and covered a period of about six weeks. Nineteen thermometers were broken during the observations by the expansion of the mercury. Girl would not have practiced deception. During the paroxysms the subjective symptoms were intense; coldness, requiring half dozen blankets, hot bags of water, etc.; nausea, and at times vomiting. She also complained of "numbness," beginning in the face and extending to the body. The objective signs were pallor and lividity of face and extremities, these appearing cold, and the body warm but not hot. The tongue was generally coated, but rarely dry. The pulse never ran over 120, and was generally under 100. The urine was normal, examined both chemically and microscopically, and nothing worthy of note found. Digestion impaired, bowels inclined to constipation, but no serious trouble; menstruated normally during the attack. At times, some tenderness in splenic and hepatic regions. At one time developed considerable tenderness in right iliac, and along the ascending colon—lasted about a week. Convalescence rapid, and she has since enjoyed perfect health.

---

TREATMENT OF PNEUMONIA.—In the *Medical Record*, December 5, Dr. A. A. Young, Newark, N. Y., commends the use of jaborandi in pneumonia. There is a relation, he says, between the urea or urates of the system and the pneumonic poison, and the severity of the malady can almost be measured by the abundance of urates present. To diminish the amount of these urates is the object of the jaborandi treatment. The drug produces, in a few minutes after its administration, flushing of the face and skin, quickly followed by profuse perspiration and intense salivation, which usually lasts from two to four hours. Drug does not depress the heart, and is slightly narcotic. Amount of urine is greatly increased, and contains urea in abundance. Method of administration is to give one-fourth grain of pilocarpine, the active principle of jaborandi. If this should not produce perspiration in one hour, one-eighth grain more is to be given. Adjuvants, as carbonate of ammonia, or sparteine, or whisky, are given, as indicated. Under this treatment the urea is eliminated, the temperature falls, the pulse and respiration are reduced in frequency and the general well-being of the patient subserved. The writer does not claim originality for this method. The reported success of this treatment in his experience for a period of six years may well encourage others to try the same plan.

---

MEDICAL PRACTICE IN CONNECTICUT.—The following spicy delineation of the medical situation in Connecticut is taken from the *Bulletin of the Connecticut Board of Health:* "SIR: Anybody can practice medicine in Connecticut. You do not need to register; you do not need a medical diploma; you do not need to know the difference between opium and peppermint; you do not, indeed, need to know anything. You can simply come and live here and begin to practice. The laws of Connecticut will sustain you in collecting your fees for professional services, if you render any which you choose to call such. But if you undertake to carry me or my trunk to the depot for pay, you must get a license. If you peddle matches or peanuts, you must get a license. If you collect the swill from your neighbors, to feed your pigs, you must get a license. If you want to empty your cesspool, you must get a license. But you can practice medicine in Connecticut *without a license.*"—*N. Y. Med. Journal.*

QUERY:—"How is it in Georgia?"

